# Influence of lumbar kyphosis and back muscle strength on the symptoms of gastroesophageal reflux disease in middle-aged and elderly people

**DOI:** 10.1007/s00586-012-2207-1

**Published:** 2012-02-28

**Authors:** Shiro Imagama, Yukiharu Hasegawa, Norimitsu Wakao, Kenichi Hirano, Nobuyuki Hamajima, Naoki Ishiguro

**Affiliations:** 1 Department of Orthopaedic Surgery, Nagoya University Graduate School of Medicine, 65, Tsurumai, Showa-ku, Nagoya, Aichi 466-8550 Japan; 2 Department of Preventive Medicine/Biostatistcs and Medical Decision Making, Nagoya University Graduate School of Medicine, 65, Tsurumai, Showa-ku, Nagoya, Aichi 466-8550 Japan

**Keywords:** Gastroesophageal reflux disease, Lumbar kyphosis, Sagittal balance, Number of drugs, Back muscle strength

## Abstract

**Objective:**

The objectives of this study was to clarify the relationship between kyphosis and Gastroesophageal reflux disease (GERD) by evaluation of spinal alignment, obesity, osteoporosis, back muscle strength, intake of oral drugs, and smoking and alcohol history in screening of a community population to determine the factors related to GERD symptoms.

**Summary of background data:**

GERD increases with age and is estimated to occur in about 30% of people. Risk factors for GERD include aging, male gender, obesity, oral medicines, smoking, and alcohol intake. It has also been suggested that kyphosis may influence the frequency of GERD, but the relationship between kyphosis and GERD is unclear.

**Subjects and methods:**

We examined 245 subjects (100 males and 145 females; average age 66.7 years old) in a health checkup that included evaluation of sagittal balance and spinal mobility with SpinalMouse^®^, GERD symptoms using the Frequency Scale for Symptoms of GERD (FSSG) questionnaire, body mass index, osteoporosis, back muscle strength, number of oral drugs taken per day, intake of nonsteroidal anti-inflammatory drugs (NSAIDs), intake of bisphosphonates, and smoking and alcohol intake.

**Results:**

Multivariate logistic regression analysis including all the variables showed that lumbar lordosis angle, sagittal balance, number of oral drugs taken per day, and back muscle strength had significant effects on the presence of GERD (OR, 1.10, 1.11, 1.09 and 1.03; 95%CI, 1.03–1.17, 1.02–1.20, 1.01–1.18 and 1.01–1.04; *p* = 0.003, 0.015, 0.031 and 0.038, respectively). The other factors showed no association with GERD.

**Conclusion:**

This study is the first to show that lumbar kyphosis, poor sagittal balance; increased number of oral drugs taken per day, and decreased back muscle strength are important risk factors for the development of GERD symptoms. Thus, orthopedic surgeons and physicians should pay attention to GERD in elderly patients with spinal deformity.

## Introduction

Gastroesophageal reflux disease (GERD) is a clinical entity that encompasses all the manifestations of exposure of the esophagus to gastric acid, and is characterized by typical and specific symptoms of heartburn and acid regurgitation. GERD increases with age and is estimated to occur in about 30% of people [1–4]. Recently, a large survey showed an increased prevalence of GERD of 37.6% in the Japanese population [5]. GERD has a significant negative impact on quality of life (QOL) [6], and this is important since independence and improvement of QOL of the elderly is needed with the recent aging of society.

Risk factors for GERD include aging, male gender, obesity, oral medicines, smoking, and alcohol intake [7–12]. It has also been suggested that kyphosis may influence the frequency of GERD [13–15], but the relationship between kyphosis and GERD is unclear. GERD is of concern in elderly patients because many of these patients have kyphosis and take oral medication. Thus, GERD in elderly patients with spinal deformity may be a particular concern in spine surgery. Therefore, the objective of this study was to clarify these issues by evaluation of spinal alignment, obesity, osteoporosis, back muscle strength, intake of oral drugs, and smoking and alcohol history in screening of a community population to determine the factors related to GERD symptoms.

## Subjects and methods

The subjects were healthy volunteers who attended a basic health checkup supported by a local government in 2009. The current study was performed in 245 subjects (100 males and 145 females) who received examinations with lumbar lateral standing radiographs, SpinalMouse^®^ (Idiag, Volkerswill, Switzerland) for sagittal balance and spinal mobility, and the Frequency Scale for Symptoms of GERD (FSSG) questionnaire for the prevalence of GERD symptoms [16]. The average age of the subjects was 66.7 years old (range: 45–91 years old). Patients under the treatment for esophageal, gastric and duodenal intestinal disease or with a surgical history involving these diseases were excluded from the study. Patients with fresh vertebral compression fracture and those with a history of spine surgery were also excluded. SpinalMouse^®^ data, GERD symptoms, body mass index (BMI), osteoporosis, back muscle strength, number of oral drugs taken per day, intake of nonsteroidal anti-inflammatory drugs (NSAIDs), intake of bisphosphonates, and smoking and alcohol intake were examined as described below. Correlations of the lumbar lordosis angle and sacral inclination angle with lumbar radiograph findings were examined to confirm the reproducibility of SpinalMouse^®^ measurements. Diagnosis of osteoporosis was based on the criteria proposed by the Japanese Society for Bone and Mineral Research [17], and was defined as a percentage of the young adult mean (%YAM) <70% in the calcaneus. The study was approved by the Committee on Ethics in Human Research of Nagoya University.

### Evaluation using SpinalMouse^®^

**Fig. 1:**
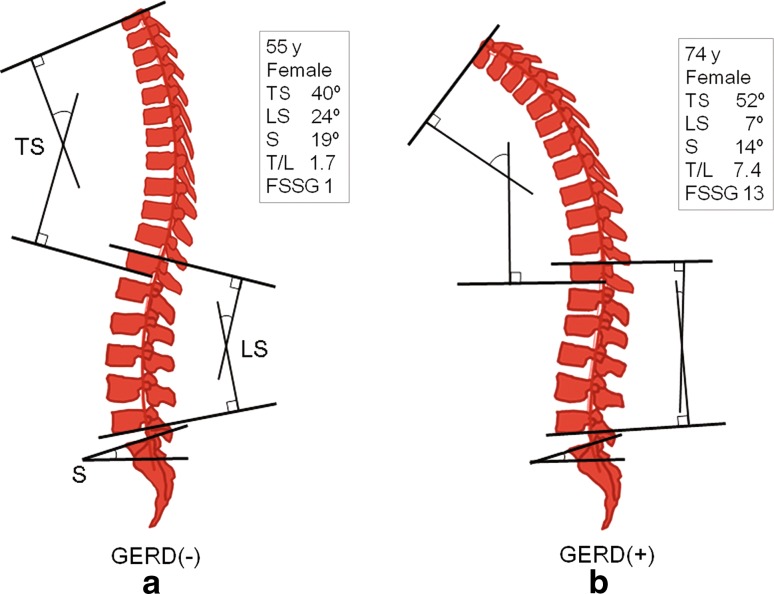
Representative SpinalMouse findings (neutral standing position). **a** The thoracic kyphosis angle (T1–T12), lumbar lordosis angle (T12–L5), and sacral inclination angle were measured by SpinalMouse. Spinal ROM was also calculated in the maximum bending position and maximum extension position. **b** GERD(+) subjects tended to have decreased lumbar lordosis followed by poor sagittal balance (that is, a larger T/L ratio) in a neutral standing position. *TS thoracic kyphosis angle, *LS* lumbar lordosis angle, *S* sacrum inclination angle, *T*/*L* thoracic/lumbar angle ratio, GERD gastroesophageal reflux disease, FSSG Frequency Scale for Symptoms of GERD

Spinal range of motion (ROM) and spinal angle were measured using SpinalMouse^**®**^, which is an electronic computer-aided device that measures sagittal spinal ROM and intersegmental angles noninvasively using the so-called surface technique. Intraclass coefficients of 0.92–0.95 have been determined for curvature measurement with SpinalMouse^**®**^ [18]. In the current study, each angle was measured three times in a neutral standing position, maximum bending position, and maximum extension position, and average data were used. The evaluation items included the thoracic kyphosis angle (T1–T12), lumbar lordosis angle (T12–L5), sacral inclination angle, thoracic spinal ROM, lumbar spinal ROM, and total spinal ROM (Fig. 1a). The thoracic kyphosis and lumbar lordosis angles are expressed as positive values in this study. Evaluation of SpinalMouse^®^ data revealed significant correlations with lumbar radiographic data for the lumbar lordosis angle (*r* = 0.791, *p* < 0.0001, SpinalMouse^®^ 20.3 ± 13.9° [mean ± standard deviation (SD)], radiograph 24.1 ± 12.7°) and sacral inclination angle (*r* = 0.645, *p* < 0.0001, SpinalMouse^®^ 9.1 ± 9.4°, radiograph 15.3 ± 7.9°). There was a tendency for the SpinalMouse^®^ angles to be smaller, but these significant correlations confirm the reliability of the SpinalMouse^®^ measurements of these angles. Therefore, the SpinalMouse^®^ data were used in further analysis. The value obtained by dividing the thoracic kyphosis angle (neutral position) by the lumbar lordosis angle (neutral position), a marker of posture with the head bent forward, was defined as the thoracic/lumbar angle ratio (T/L ratio) and used as an index of sagittal balance [19–22]. Elderly persons with a large T/L ratio are likely to lean forward because thoracic kyphosis is not compensated by lumbar lordosis [19]. Strictly, examination of the whole spine radiograph is required to evaluate sagittal balance, but this cannot be achieved in a basic health checkup. Therefore, we used SpinalMouse^®^ for evaluating spinal balance.

### Evaluation of GERD

All patients were asked to respond to the FSSG questionnaire, a simple questionnaire that has recently been developed by Kusano et al. [16]. The FSSG questionnaire is appropriate for management of GERD in general practice patients; endoscopy is not required, but the results from the FSSG questionnaire correlate strongly with endoscopic findings. The questionnaire is a self-reported instrument that contains 12 questions and is written in simple and easy-to-understand language. The following definitions are used in the FSSG questionnaire to identify symptoms: (1) Do you get heartburn? (2) Does your stomach get bloated? (3) Does your stomach ever feel heavy after meals? (4) Do you sometimes subconsciously rub your chest with your hand? (5) Do you ever feel sick after meals? (6) Do you get heartburn after meals? (7) Do you have an unusual (e.g., burning) sensation in your throat? (8) Do you feel full while eating meals? (9) Does food get stuck when you swallow? 10) Do you get bitter liquid (acid) coming up into your throat? (11) Do you burp a lot? and (12) Do you get heartburn if you bend over? Symptom frequency was measured on a scale of never = 0; occasionally = 1; sometimes = 2; often = 3; and always = 4. GERD was diagnosed based on a FSSG score of ≥8 points [14, 16, 23] and subjects were divided into GERD(+) and GERD(−) groups.

### Back muscle strength

**Fig. 2:**
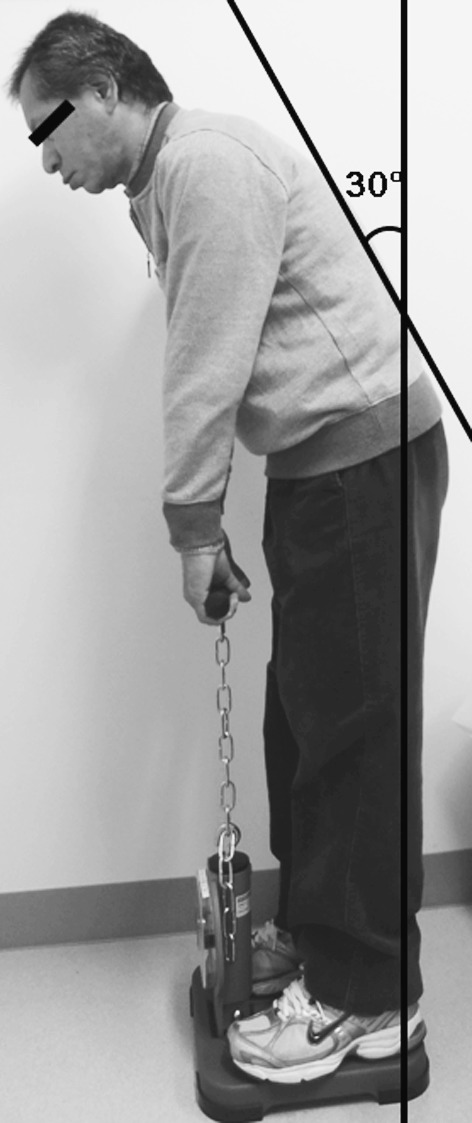
Back muscle strength was determined in a standing posture with 30° lumbar flexion using a back muscle strength meter

Back muscle strength was determined from the maximal isometric strength of the trunk muscles in a standing posture with 30° lumbar flexion using a back muscle strength meter (T.K.K.5002, Takei Co., Japan) (Fig. 2) [19, 24, 25]. The average force from two trials was recorded. The maximum strength in each trial was measured and these values showed high reproducibility (*r* = 0.990, *p* < 0.0001). All subjects were assessed by one examiner who was blinded to the results of other evaluations.

### Interview concerning selected characteristics

Physical characteristics were determined by experienced interviewers using a questionnaire at the time of the Comprehensive Health Examination. Information on gender, age, BMI, FSSG questionnaire, number of oral drugs taken per day, intake of NSAIDs, intake of bisphosphonates, history of spine surgery, history of alcohol intake, and smoking history were obtained. The responses for alcohol intake were used to divide the subjects into those with a history of alcohol intake and those who had never drank alcohol. Information for smoking history was treated in the same manner. Support was provided so that the subjects could answer all questions by themselves.

### Statistical analysis

All data are shown as means ± SD. Correlations between variables were analyzed using Pearson correlation coefficient analysis and simple regression analysis. An unpaired *t* test and Chi-square test was used to evaluate differences between the GERD(+) and GERD(−) groups. Univariate and multivariate logistic regression analyses were performed to evaluate the odds ratio (OR) with 95% confidence interval (95%CI) for potential risk factors for GERD. Probability values of less than 0.05 were considered to be statistically significant.

## Results

The mean values of measured variables in the subjects are listed in Table 1 and correlations between FSSG scores and other variables are shown in Table 2. The FSSG score was negatively correlated with the lumbar lordosis angle and back muscle strength; and positively correlated with the T/L ratio and the number of oral drugs taken per day (Table 2). The 245 patients were classified into two groups based on GERD symptoms, with 60 (24.5%) in the GERD(+) group (Table 3). The lumbar lordosis angle, back muscle strength and sacral inclination angle were significantly smaller and the T/L ratio and the number of oral drugs taken per day were significantly larger in the GERD(+) group compared to the respective values in the GERD(−) group. Gender, age, BMI, osteoporosis, thoracic kyphosis angle, spinal ROM, intake of oral NSAIDs, oral bisphosphonates, and smoking and alcohol history did not differ significantly between the GERD(+) and GERD(−) groups (Table 3). Univariate and multivariate logistic regression analyses were performed to evaluate the OR for risk factors for GERD. In univariate analysis, the number of oral drugs taken per day, lumbar lordosis angle, back muscle strength, T/L ratio, and sacral inclination angle were significantly associated with the presence of GERD (Table 4). In multivariate logistic regression analysis including all the variables, lumbar lordosis angle, T/L ratio, number of oral drugs taken per day, and back muscle strength had a significant association with the presence of GERD (OR, 1.10, 1.11, 1.09 and 1.03; 95%CI, 1.03–1.17, 1.02–1.20, 1.01–1.18 and 1.01–1.04; *p* = 0.003, 0.015, 0.031 and 0.038, respectively) (Table 5). These results show that a decrease in the lumbar lordosis angle, poor sagittal balance, an increased number of oral drugs per day, and decreased back muscle strength are important risk factors for GERD.

**Table 1:** Clinical background of the subjects

Item	Value
Number	245
Male/female	100/145
Age (years)	66.7 (8.4)
BMI (kg/cm^2^)	23.6 (3.2)
BMD; YAM (%)	81.9 (14.7)
Osteoporosis (<YAM 70%)	52 (21.2%)
SpinalMouse	
Thoracic kyphosis angle (°)	38.2 (12.2)
Lumbar lordosis angle (°)	20.3 (13.9)
Sacral inclination angle (°)	9.1 (9.4)
T/L ratio	2.4 (4.7)
Thoracic spinal ROM (°)	24.0 (16.7)
Lumbar spinal ROM (°)	42.7 (17.8)
Total spinal ROM (°)	66.7 (21.0)
GERD	
FSSG score	4.9 (5.4)
GERD(+) (FSSG score ≥8)	60 (24.5%)
Back muscle strength (kg)	77.9 (30.7)
Number of oral drugs taken per day	3.7 (4.9)
Intake of NSAIDs	22 (9.1%)
Intake of bisphosphonates	19 (7.8%)
Smoker	27 (11.0%)
Alcohol drinker	108 (44.1%)

Values are shown as the number of patients (percentage in parentheses) or as the mean (SD)

*BMI* body mass index, *BMD* bone mineral density, *YAM* young adult mean, *T/L ratio* thoracic/lumbar angle ratio, *ROM* range of motion, *GERD* gastroesophageal reflux disease, *FSSG* Frequency Scale for Symptoms of GERD, *NSAIDs* nonsteroidal anti-inflammatory drugs

**Table 2:** Correlation between total FSSG score and other variables

Variables	Coefficient (*r*)	Significance (*p*)
**T/L ratio**	**0.152**	0.023*
**Back muscle strength (kg)**	**−0.148**	0.027*
**Lumbar lordosis angle (°)**	**−0.141**	0.036*
**Total intake of oral drugs per day**	**0.140**	0.036*
Age (years)	−0.034	0.611
BMI (kg/cm^2^)	0.003	0.960
BMD; YAM (%)	−0.001	0.983
Thoracic kyphosis angle (°)	0.126	0.061
Sacral inclination angle (°)	−0.079	0.240
Thoracic spinal ROM (°)	−0.017	0.797
Lumbar spinal ROM (°)	0.065	0.336
Total spinal ROM (°)	0.041	0.544

Bold values are statistically significant

*T/L ratio* thoracic/lumbar angle ratio, *BMI* body mass index, *BMD* bone mineral density, *YAM* young adult mean, *ROM* range of motion, *FSSG* frequency scale for symptoms of GERD, *GERD* gastroesophageal reflux disease

* Significant correlation (*p* < 0.05). The parameters are arranged in order of significance

**Table 3:** Difference in variables between subjects with and without GERD

Variables	GERD(+) *n* = 60	GERD(−) *n* = 185	Significance (*p*)
**Total intake of oral drugs per day**	**5.5 (6.6)**	**3.2 (4.2)**	0.0015***
**Lumbar lordosis angle (°)**	**13.8 (19.1)**	**22.4 (11.2)**	0.0029***
**T/L ratio**	**3.6 (5.1)**	**2.0 (3.2)**	0.009**
**Back muscle strength (kg)**	**69.4 (32.5)**	**80.4 (29.8)**	0.016*
**Sacral inclination angle (°)**	**7.2 (11.2)**	**10.0 (8.5)**	0.046*
Female (%)	60.0 (*n* = 36)	57.3 (*n* = 106)	>0.999
Age (years)	66.8 (9.3)	66.4 (8.2)	0.790
BMI (kg/cm^2^)	23.8 (3.1)	23.5 (3.2)	0.452
BMD; YAM (%)	79.7 (17.0)	82.7 (13.9)	0.183
Osteoporosis (<YAM 70%)	30.0 (*n* = 18)	18.4 (*n* = 34)	0.141
Thoracic kyphosis angle (°)	35.7 (15.3)	38.4 (10.5)	0.119
Thoracic spinal ROM (°)	23.4 (19.6)	24.3 (15.7)	0.725
Lumbar spinal ROM (°)	43.4 (19.4)	42.7 (17.3)	0.805
Total spinal ROM (°)	66.8 (23.9)	67.0 (20.3)	0.944
Intake of NSAIDs (%)	11.7 (*n* = 7)	8.1 (*n* = 15)	0.447
Intake of bisphosphonates (%)	10.0 (*n* = 6)	7.0 (*n* = 13)	0.582
Smoker (%)	8.3 (*n* = 5)	10.8 (*n* = 20)	0.634
Alcohol drinker (%)	41.7 (*n* = 25)	44.3 (*n* = 82)	0.652

Bold values are statistically significant

Values are shown as the mean (SD or the number of patients)

*GERD* gastroesophageal reflux disease, *T/L ratio* thoracic/lumbar angle ratio, *BMI* body mass index, *BMD* bone mineral density, *YAM* young adult mean, *ROM* range of motion, *NSAIDs* nonsteroidal anti-inflammatory drugs

* Significant difference. The parameters are arranged in order of significance

**Table 4:** Results of univariate logistic regression analysis: odds ratio (OR) with 95% confidence interval (95% CI) for the risk of GERD

Parameter	OR	95% CI	Significance (*p*)
**Total intake of oral drugs per day**	**1.09**	**1.03–1.15**	0.003*
**Lumbar lordosis angle (−°)** ^**a**^	**1.03**	**1.01–1.05**	0.004*
**Back muscle strength (− kg)** ^**a**^	**1.01**	**1.00–1.02**	0.018*
**T/L ratio**	**1.06**	**1.01–1.11**	0.019*
**Sacral inclination angle (°)**	**0.97**	**0.94–0.99**	0.048*
Female	1.02	0.56–1.85	0.951
Age (years)	1.01	0.97–1.04	0.789
BMI (kg/cm^2^)	1.04	0.94–1.14	0.451
BMD; YAM (%)	0.99	0.97–1.01	0.183
Thoracic kyphosis angle (°)	0.98	0.96–1.01	0.122
Thoracic spinal ROM (°)	1.00	0.98–1.02	0.724
Lumbar spinal ROM (°)	1.00	0.99–1.02	0.804
Total spinal ROM (°)	1.01	0.99–1.02	0.165
Intake of NSAIDs	1.44	0.56–3.70	0.456
Intake of bisphosphonates	1.41	0.51–3.89	0.507
Smoker	1.36	0.49–3.80	0.559
Alcohol drinker	1.15	0.63–2.09	0.644

Bold values are statistically significant

*GERD* gastroesophageal reflux disease, *T/L ratio* thoracic/lumbar angle ratio, *BMI* body mass index, *BMD* bone mineral density, *YAM* young adult mean, *ROM* range of motion, *NSAIDs*: nonsteroidal anti-inflammatory drugs

* Significant difference. The parameters are arranged in order of significance

^a^ For easy comprehension, the ORs for the lumbar lordosis angle and back muscle strength are shown using negative units

**Table 5:** Results of multivariate logistic regression analysis: odds ratios (OR) with 95% confidence interval (95% CI) for the risk of GERD

Parameter	OR	95% CI	Significance (*p*)
**Lumbar lordosis angle (−°)** ^**a**^	**1.10**	**1.03–1.17**	0.003*
**T/L ratio**	**1.11**	**1.02–1.20**	0.015*
**Total intake of oral drugs per day**	**1.09**	**1.01–1.18**	0.031*
**Back muscle strength (− kg)** ^**a**^	**1.03**	**1.01–1.04**	0.038*
Female	1.02	0.56–1.85	0.951
Age (years)	0.96	0.91–1.01	0.104
BMI (kg/cm^2^)	1.05	0.95–1.18	0.346
BMD; YAM (%)	0.98	0.96–1.01	0.216
Thoracic kyphosis angle (°)	1.01	0.98–1.05	0.482
Sacral inclination angle (°)	1.07	0.99–1.17	0.099
Thoracic spinal ROM (°)	1.00	0.98–1.02	0.955
Lumbar spinal ROM (°)	1.02	0.99–1.05	0.076
Total spinal ROM (°)	1.01	0.99–1.03	0.148
Intake of NSAIDs	2.45	0.63–9.54	0.197
Intake of bisphosphonates	1.19	0.32–4.50	0.794
Smoker	1.07	0.32–3.62	0.915
Alcohol drinker	0.91	0.40–2.05	0.810

Bold values are statistically significant

*GERD* gastroesophageal reflux disease, *T/L ratio* thoracic/lumbar angle ratio, *BMI* body mass index, *BMD* bone mineral density, *YAM* young adult mean, *ROM* range of motion, *NSAIDs* nonsteroidal anti-inflammatory drugs

* Significant difference. The parameters are arranged in order of significance

^a^For easy comprehension, the ORs for the lumbar lordosis angle and back muscle strength are shown using negative units

## Discussion

Regurgitation of gastric contents into the esophagus is prevented by the lower esophageal sphincter (LES). GERD is induced by decreased LES pressure, and this may be caused by esophageal hiatal hernia, which affects the function of the anti-reflux barrier at the gastroesophageal junction [26, 27]. The prevalence of hiatal hernia increases in elderly female patients [7] and the presence of hiatal hernia has been correlated with the incidence of GERD [28, 29]. This partly accounts for the increased prevalence of GERD with the aging of society.

Osteoporosis and kyphosis have also been suggested to contribute to the increased prevalence of GERD and hiatal hernia [12, 13]. Yamaguchi et al. found that the presence and number of vertebral fractures were significantly associated with hiatal hernia in 87 postmenopausal women [12], and Kusano et al. showed that the size of hiatal hernia had a positive correlation with the severity of kyphosis assessed using a digital camera in 100 elderly women [13]. However, these reports lacked detailed information on kyphosis, such as the location of the kyphosis (thoracic or lumbar), kyphosis angle, and sagittal balance. Recently, Miyakoshi et al. examined the impact of spinal kyphosis on GERD using plain radiographs in 112 patients (3 males and 119 females) with osteoporotic vertebral fractures, and found that an increase in lumbar kyphosis angle and number of lumbar vertebral fractures are important risk factors for GERD in osteoporotic patients [14]. However, most of these patients were osteoporotic women, and factors such as BMI, back muscle strength, smoking and alcohol intake were not examined. Therefore, most previous reports have not examined all the potential factors associated with GERD. Thus, the current report is the first to evaluate the association of GERD with age, gender, obesity, osteoporosis, kyphosis angle, spinal ROM, spinal alignment, muscle strength, drug intake, smoking and alcohol intake.

**Fig. 3:**
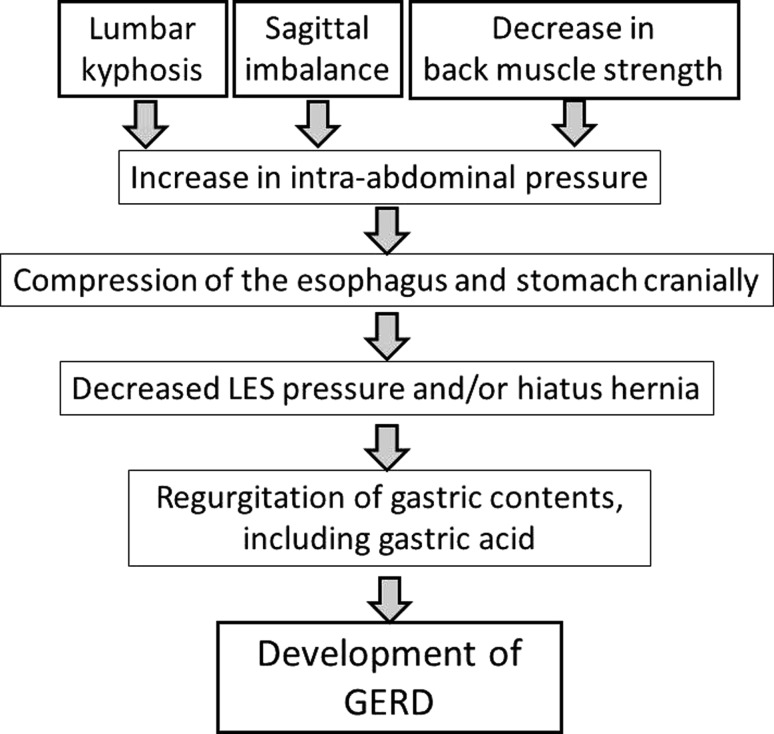
Proposed mechanism of occurrence of GERD due to spinal disorder. Lumbar kyphosis, sagittal imbalance, and decrease in back muscle strength may independently or synergistically increase intra-abdominal pressure, with a subsequent decrease of LES pressure and hiatus hernia leading to regurgitation of gastric acid and finally causing GERD

This study revealed that a decrease of the lumbar lordosis angle, poor sagittal balance, an increased number of oral drugs, and decreased back muscle strength are important risk factors for GERD (Fig. 1b). Reduced lumbar lordosis was revealed as the most significant risk factor for development of GERD symptoms, which is consistent with the results of Miyakoshi et al. [14]. In our multivariate analysis, every additional 1° of lumbar kyphosis increased the chance of having GERD by approximately 1.1, indicating a 2.59 times higher risk of GERD development with a decrease of lumbar lordosis of 10°. In contrast, the angle of thoracic kyphosis was not an important factor. These data clearly show that GERD is significantly associated with kyphosis of the lumbar spine, but not of the thoracic spine. We suggest that the mechanism may involve an increase in intra-abdominal pressure caused by lumbar kyphosis [15], with subsequent compression of the esophagus and stomach cranially. These changes may then induce decreased LES pressure and hiatus hernia, leading to regurgitation of gastric contents, including gastric acid, and finally causing GERD (Fig. 3).

We also found that the T/L ratio was an independent risk factor for development of GERD symptoms in multivariate analysis. This and the fact that thoracic kyphosis was not a risk factor for GERD indicates that global sagittal balance has an important relationship with development of GERD symptoms, as well as local spinal alignment. As we found in a previous study, most elderly people cannot maintain sagittal balance due to inflexibility of the spine, which results in decompensated sagittal balance with an increased T/L ratio [19]. Global sagittal balance should be somewhat influenced by lumbar lordosis, but this result suggests that compression of the abdomen due to global sagittal imbalance may be a cause of GERD, even if a decrease of lumbar lordosis angle is not accompanied by global sagittal imbalance. Shimizu et al. [30] conducted spinal fusion from the thoracic spine to the sacrum in a 72-year-old female with poor spinal alignment and showed that GERD improved immediately after surgery, and we have also experienced a similar surgical case in a 69-year-old female in our university hospital (she is not one of the patients in this study). Such cases support the proposal of a relationship between global spinal alignment and GERD, and suggest that spinal surgery may be used to treat both kyphosis and GERD in patients with severe back pain and reduce dietary intake. Patients with ankylosing spondylitis, rheumatoid arthritis, Parkinson disease, myopathies and other diseases with neuromuscular deficiency were excluded in this study, but these diseases also influence global spinal alignment and thus are likely to contribute to gastroesophageal motility problems. Therefore, it is important clinically to consider the increased risk of GERD in such patients.

The FSSG score had a significant correlation with the number of oral drugs taken per day, but showed no relationship with oral administration of NSAIDs or bisphosphonates. Miyakoshi et al. also found that the total number of drugs, but not oral NSAIDs or bisphosphonates, was significantly associated with the incidence of GERD [14]. Thus, drug-induced upper gastrointestinal pathology [31, 32] and an association with GERD appear to be common in elderly patients who take many oral medications. The dose and duration of administration of NSAIDs may also be important in development of GERD, and the absence of this association in the current study may be due to the irregular daily intake of NSAIDs. For bisphosphonates, alendronate therapy was found not to contribute to upper gastrointestinal pathology in a large epidemiological study [33], consistent with our current findings. We also note that our subjects were relatively healthy and few were taking these drugs, so care is required in extrapolation of these findings. However, we believe that there is a relationship between total drug intake and GERD.

The current study is the first to show a relationship between reduced back muscle strength and the incidence of GERD. Our previous cohort study showed that improved back muscle strength was an important factor for maintaining the lumbar lordosis angle and spinal sagittal balance [19]. Therefore, it is likely that decreased back muscle strength will lead to lumbar kyphosis, a bent-forward posture and an increase in intra-abdominal pressure, resulting in GERD. Multivariate logistic regression analysis indicated that weakness of muscle strength may have a direct influence on GERD, independent from an interrelationship with lumbar kyphosis. However, the relationship between GERD and muscle strength has not been reported previously and we did not examine the relationship between abdominal muscle strength and back muscle strength in this study. If the relationship between muscle strength and GERD can be confirmed in further studies, appropriate muscle training may be useful for prevention of GERD.

We used data obtained from SpinalMouse^®^ and the FSSG in the current study. SpinalMouse^®^ provides a simple and noninvasive test that has been shown to be reliable in many reports [18, 19, 25, 34–36]. In this study, SpinalMouse^®^ data showed a significant correlation with findings from lumbar radiographs, although there was a tendency for the SpinalMouse^®^ measurements to give angles that were smaller than those on plain radiographs. However, the reliability of the test decreases in persons with thick soft tissues and for lower lumbar vertebra data. SpinalMouse^®^ measurements are also difficult in patients with surgical resection of the spinous process. Therefore, we should not think that we can use the SpinalMouse^®^ angle instead of radiographic findings completely. However, SpinalMouse^®^ is suitable for ROM evaluation based on differences in measurement and comparison of alignments, and can be used for evaluation of total spinal mobility over a short period of time with no side effects. In this health checkup, the cost was supported by a small local government and it would be expensive to add more radiographic examinations and facilities for plain radiographs. Furthermore, the health checkup includes internal medicine, urology, ophthalmology and otolaryngology tests, which limits the time and patience of subjects available for more radiographic assessments. For these reasons of expense and time, it is difficult to evaluate a functional radiograph or a whole spine radiograph in this kind of health checkup. Thus, SpinalMouse^®^ is a useful tool for measurement of spinal mobility and alignment. The FSSG is a simple questionnaire based on symptoms that has been shown to correlate strongly with endoscopic findings [16]. Strictly, diagnosis of GERD requires endoscopy, but performance of endoscopy is also difficult in a health checkup, and many patients with GERD show no evidence of the disease on endoscopy [37–39]. In contrast, use of the FSSG questionnaire may fail to identify asymptomatic cases of GERD. However, we believe that all patients with symptomatic GERD can be identified using this questionnaire, and that the FSSG is a useful noninvasive tool for evaluation of GERD in community screening and orthopedic clinics.

There are several other limitations of this study. First, this study was performed in one ethnic group, and inclusion of other ethnicities is needed in a future study. Also, the percentage of patients with osteoporosis and those treated with oral drugs was small because the subjects were relatively healthy. Thus, we need more subjects to clarify the relationships of GERD with smoking and alcohol intake. However, the current study is of value as the first examination of the association of GERD with multiple clinical factors in healthy subjects. Our results showed that lumbar kyphosis, poor spinal balance, increased number of oral drugs taken per day, and decreased back muscle strength are important risk factors for development of GERD symptoms. These findings suggest that GERD may be prevented by maintaining spinal balance and back muscle strength in elderly patients. This indicates that orthopedic surgeons should pay careful attention to GERD in elderly patients with spinal deformity.
